# Unveiling the sleep‐cardiovascular connection: Novel perspectives and interventions—A narrative review

**DOI:** 10.1002/hsr2.1773

**Published:** 2023-12-15

**Authors:** Christin Berjaoui, Bethlehem Tesfasilassie Kibrom, Mohammad Ghayyad, Safaa Joumaa, Nihal Talal Al Labban, Abubakar Nazir, Charbel Kachouh, Abdulrahmon Akanmu Moradeyo, Magda Wojtara, Olivier Uwishema

**Affiliations:** ^1^ Department of Medicine Oli Health Magazine Organization, Research, and Education Kigali Rwanda; ^2^ Department of Medicine, Faculty of Medicine Beirut Arab University Beirut Lebanon; ^3^ Department of Medicine, College of Health Sciences Addis Ababa University Addis Ababa Ethiopia; ^4^ Department of Medicine, Faculty of Medical Science Lebanese University Beirut Lebanon; ^5^ Department of Medicine King Edward Medical University Lahore Pakistan; ^6^ Department of Medicine Saint‐Joseph University Beirut Lebanon; ^7^ Department of Medicine and Surgery Ladoke Akintola University of Technology Ogbomosho Nigeria; ^8^ Department of Medicine Clinton Global Initiative University New York New York USA; ^9^ Department of Medicine Karadeniz Technical University Trabzon Turkey

**Keywords:** cardiovascular risks, sleep, sleep biomarkers, sleep disorder, sleep technology, sleep therapy

## Abstract

**Introduction:**

Sleep is an important neurophysiological condition that is intricately linked to general health, laying the basis for both physiological and psychological well‐being. A thorough examination of sleep disorders and cardiovascular health demonstrates their deep relationship, emphasizing the numerous diagnostic tools and treatment techniques available.

**Aim:**

This study aims to examine the impact, mechanisms, diagnostic techniques, treatment strategies, implications, and healthcare interventions of the sleep‐cardiovascular connection, to better understand the relationship between sleep disorders and cardiovascular health.

**Methods:**

The paper reviews key studies conducted from 2015‐till date, investigating the impact of sleep disorders on the cardiovascular system. It looked into data relating to cardiovascular outcomes based on the degree of sleep disorders, considered potential confounding factors, and addressed current research constraints.

**Results:**

The findings highlight a strong link between sleep problems and poor cardiovascular outcomes. Emerging diagnostic tools, such as enhanced sleep‐related technology and biomarkers, open up new avenues for determining the impact of sleep disturbances on cardiovascular health. In addition, the research discusses several treatment options, ranging from cognitive behavioral therapy to pharmaceutical therapies, and their potential benefits in addressing sleep‐related cardiovascular risks.

**Conclusion:**

The complex association between sleep disturbances and cardiovascular health emphasizes the need to recognize sleep as a critical component of overall well‐being. Thus collaboration among medical disciplines, as well as individualized therapies, are critical to improving patient care. Moreover, Understanding and managing the consequences of sleep problems on cardiovascular health can lead to more effective interventions, better outcomes, and improved public health as research advances.

## 1 INTRODUCTION

Sleep is an essential neurophysiological condition that is intricately linked to overall health and serves as a foundation for both physiological and psychological strength.^1^ Different features of sleep can be evaluated by the number of hours slept or precisely (such as short or long sleepers), and based on disorders (such as insomnia, sleep apnea, or restless leg syndrome).^2^ The American Academy of Sleep Medicine (AASM) and the Sleep Research Society (SRS) collaborated to construct a consensus panel with the goal of establishing guidelines for optimal sleep duration.^3^ Simultaneously, the National Sleep Foundation (NSF) and the American Thoracic Society (ATS) also developed their own comprehensive guideline sheet and a companion manuscript on sleep length. All of these programs came to the same conclusion: people need at least 7 h of sleep per night to maintain peak health and functionality (the NSF statement also proposed an upper limit of 9 h).^3^, ^4^ It has been shown that sleep duration and disturbances differ between countries and regions, and sleep is affected by cultural factors, such as cultural values, beliefs, and practices.^5^ Based on findings from the NSF's survey, it's evident that merely 48% of adults in the United States maintain a consistent sleep duration, while 26% typically sleep between 6 and 7 h each night, and 20% get less than 6 h of sleep per night.^6^ In 2016, an online national survey in Australia revealed the mean sleep time to be 7 h, while 8% slept more than 9 h, and 12% slept less than 5½ h. This survey also showed that in 2016, inadequate sleep quality affected 45% of adults in Australia.^7^

Moreover, according to data on Dutch adults, 32.1% had a problem related to poor sleep quality, and 43.2% reported not getting enough sleep.^8^ Another study on three preindustrial societies illustrated that, on average, hunters and gatherers in Africa and Bolivia have a sleep duration of 5.4–7.1 h.^9^

A growing percentage of people are limiting their sleep due to rising demands and lifestyle modifications such as extended work hours, increased environmental lighting, and the inclusion of new communication devices that create a lifestyle of perpetual availability.^10^, ^11^ However, these shifts in sleep patterns aren't without repercussions. Straying from the recommended sleep duration could significantly endanger health, as the extent of the adverse effects of irregular sleep on physical and mental well‐being are only just starting to be uncovered.^12^

Sleep deprivation and sleep disorders ‐insomnia, sleep apnea, and restless leg syndrome‐ are widespread health concerns that hold a strong link with cardiovascular health through shared physiological mechanisms.^13^ These issues can be effectively managed using nonpharmacological interventions.^10^ Research has consistently indicated that self‐reported inadequate sleep duration and sleep disorders contribute to adverse health outcomes like obesity, cardiovascular diseases, and mortality.^4^, ^10^ However, despite this, only a limited number of studies have explored individual variations in responses to insufficient sleep and the actual physiological sleep requirements, as well as their associations with cardiovascular health consequences.^14^ Compelling evidence suggests that the psychosocial factors influencing susceptibility or resilience to cardiovascular diseases are also interconnected with sleep patterns.^15^ Remarkably, there exists a bidirectional relationship between sleep disorders and cardiovascular ailments. Sleep disorders heighten the risk of cardiovascular problems, while pre‐existing cardiovascular issues can likewise lead to disturbances in sleep patterns.^16^

Specific sleep problems, such as sleep apnea, have been shown to cause systemic hypertension and potentially precipitate illnesses such as myocardial infarction and congestive heart failure. If left untreated, this cascade of effects can increase the risk of heart disease, stroke, and even death.^17^, ^18^ Conversely, cardiovascular disease can impair sleep, as seen by patients experiencing paroxysmal nocturnal dyspnea (PND) due to Cheyne‐Stokes’ breathing pattern.^19^ To lessen the burden of these sleep problems, a better understanding of the mechanisms that underpin cardiovascular illnesses is required. As a result, addressing sleep disturbances has the potential to reduce the risk of acquiring or aggravating cardiovascular issues.^20^

## 2 METHODOLOGY

### 2.1 Selection criteria and data collection

This narrative review offers a comprehensive framework to provide a comprehensive sight into the complex link between sleep and cardiovascular health, giving insights into pathophysiological causes, clinical consequences, and prospective therapeutic approaches. The inclusion criteria were full text articles in English that were only published between 2015 and 2023. This timeframe was chosen to accommodate the latest development. A thorough literature search was undertaken utilizing the PubMed, Scopus, and Google Scholar databases. The terms used in the search were “sleep,” “sleep disorder,” “cardiovascular Risks,” “sleep technology,” “sleep biomarkers,” “sleep therapy,” “stroke,” and “hypertension.” Our search strategy also benefited from manually searching through relevant guidelines from the American Academy of Sleep Medicine (AASM) and the Sleep Research Society (SRS). We were strictly adherent to our exclusion criteria to increase the likelihood of producing reliable and reproductive results and reduce possible bias. The exclusion criteria were studies that are not case reports, studies which were not written in English, and ones that did not report outcomes, conference papers, posters and unpublished or non‐peer‐reviewed studies. Furthermore, our study worked with a meshwork of around 529 articles where we considered 165 and used 68 in our study. We selected these articles for the avoidance of redundancy, they were more focused on the aim of our article, and they did not focus on a specific gender, age, race and color. A well‐aimed overview of the employed methodology is presented in Table 1.

**Table 1:** Summary of the methodology

Methodology steps	Description
**Literature search**	PubMed, Scopus, and Google Scholar databases.
**Inclusion criteria**	Full‐text articles in English published between 2015 and 2023 that focused on history of sleep disturbances, including sleep disturbances, hypertension, and cardiovascular health.
**Exclusion criteria**	Case reports, Studies not written in English, Studies that do not report outcome, Conference papers, Posters, Unpublished and non‐peer‐reviewed studies
**Search terms**	Keywords such as “sleep,” “sleep disorder,” “cardiovascular Risks,” Additional keywords include, “sleep technology,” “sleep biomarkers,” “sleep therapy,” “stroke,” and “hypertension.”
**Additional examination**	Manual search was conducted to include and consider relevant guidelines from the American Academy of Sleep Medicine (AASM) and the Sleep research Society (SRS)

## 3 SLEEP DISORDERS AND ITS MECHANISMS IMPACT ON CARDIOVASCULAR HEALTH

Insomnia can be defined as a problem starting or sustaining sleep that is related to daytime impairment.^21^ Sleep issues must occur at least three times each week and last for at least 3 months to be chronic insomnia otherwise it is short term.^21^

Dysesthesia in the legs, which is typically described as bubbly or a “creepy and crawly” sensation, tingling, tickling, merely general restlessness, or stretching sensation in the leg muscles that occurs during rest and is relieved by activity, are characteristics of restless legs syndrome (RLS). According to Indian research, most patients encounter one of the following four strategies: moving their legs, getting up from the bed and walking, getting someone to massage their legs, or tying a rope around their legs. Nearly all these problems occur at night.^22^ OSA is defined by repeated collapsing of the upper airway during sleep, which leads to recurring drops in oxygen levels, awakenings during the night, and disrupted sleep patterns.^23^

The mechanism of collapsing is generally unrevealed but obesity, craniofacial abnormalities, alterations in upper airway muscle function, pharyngeal neuropathy, and fluid shift towards the neck all can contribute.^24^

Individuals might display typical signs such as snoring, feeling unrested upon waking, sudden awakenings due to choking, diminished sleep quality, impaired cognitive functioning, and even instances of brief unconsciousness while driving, resulting in minor accidents. Less frequent than OSA, central sleep apnea (CSA) involves apneic episodes where breathing stops without corresponding respiratory effort.^23^

It can be primary (idiopathic), or secondary associated with Cheyne‐Stokes respiration (CSR), drugs, certain medical disorders including chronic renal failure, or after receiving positive airway pressure (PAP) a therapy for OSA. Risk factors for CSA are age, heart failure, male gender, and chronic opioid usage. Moreover, severe OSA and mixed central obstructive PSG episodes consist of additional risk factors for emergent treatment.^25^

**Figure 1:**
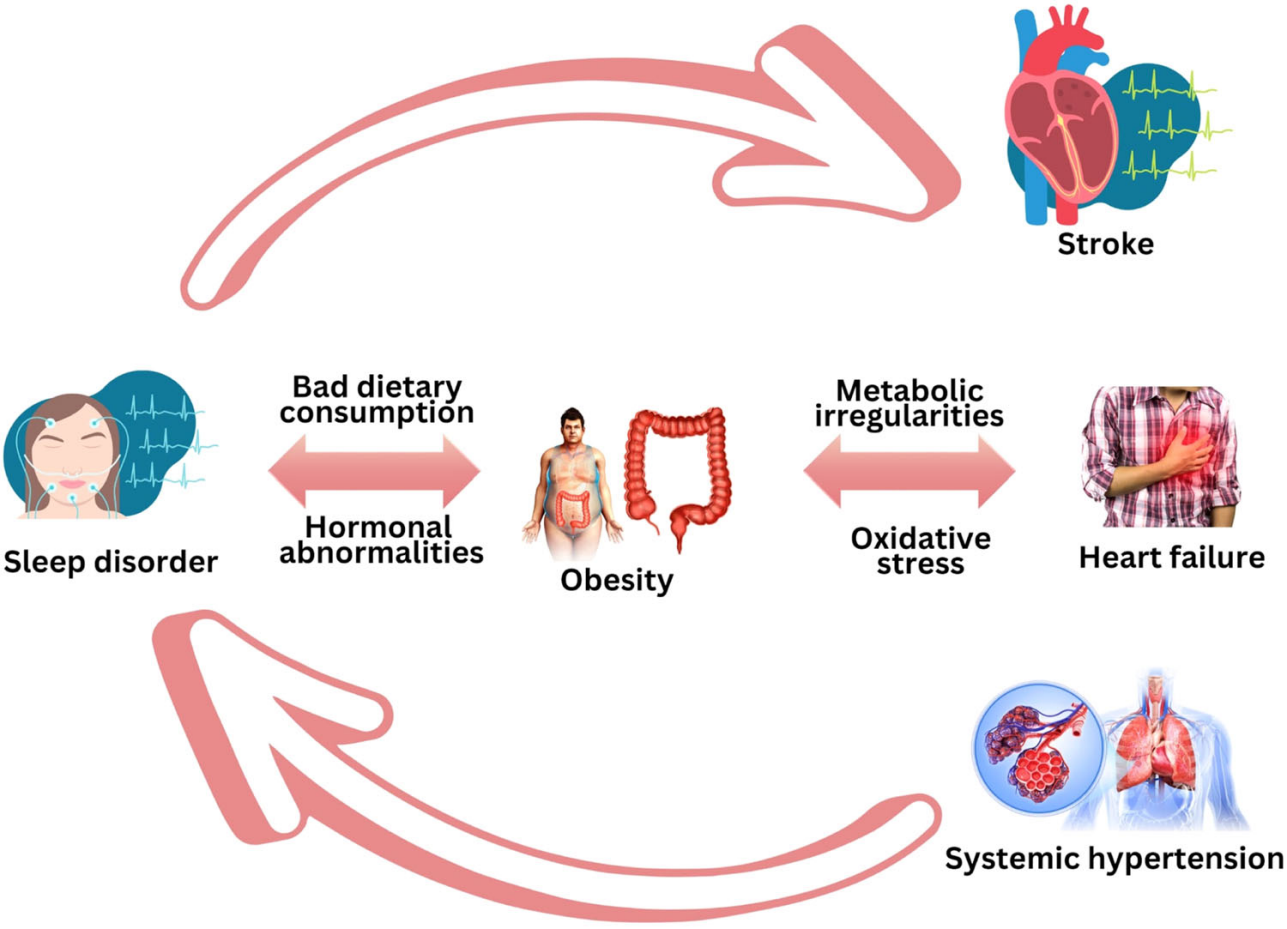
Figure 1 shows the relationship between sleep disorders and cardiovascular disease.

Research conducted in China in 2021 shows that partial sleep deprivation decreases cardiac vague activity and thus increases the heart rate.^26^ Through sympathetic over activity, sympathovagal imbalance, and arterial baroreflex, lack of sleep also raises blood pressure.^27^ In addition, dietary consumption and insufficient sleep both contribute to hormone abnormalities. Melatonin deficiency is one such imbalance that can change the metabolic circadian rhythm and increase the risk of weight gain and metabolic syndrome.^28^ The rise in food consumption due to inadequate sleep appears to be primarily influenced by pleasure‐related factors rather than hormonal elements. Because they are up for longer periods of time, short sleepers have more time and opportunity to eat. This is in line with research that reveals short sleepers eat more frequently throughout the day and snack more frequently.^29^ Numerous widespread disorders, including cardio metabolic, neurodegenerative, chronic pain, and autoimmune diseases, are prospectively linked to an elevated risk of sleep disruptions, including interrupted sleep and short sleep duration.^30^ (Figure 1).

**Figure 2:**
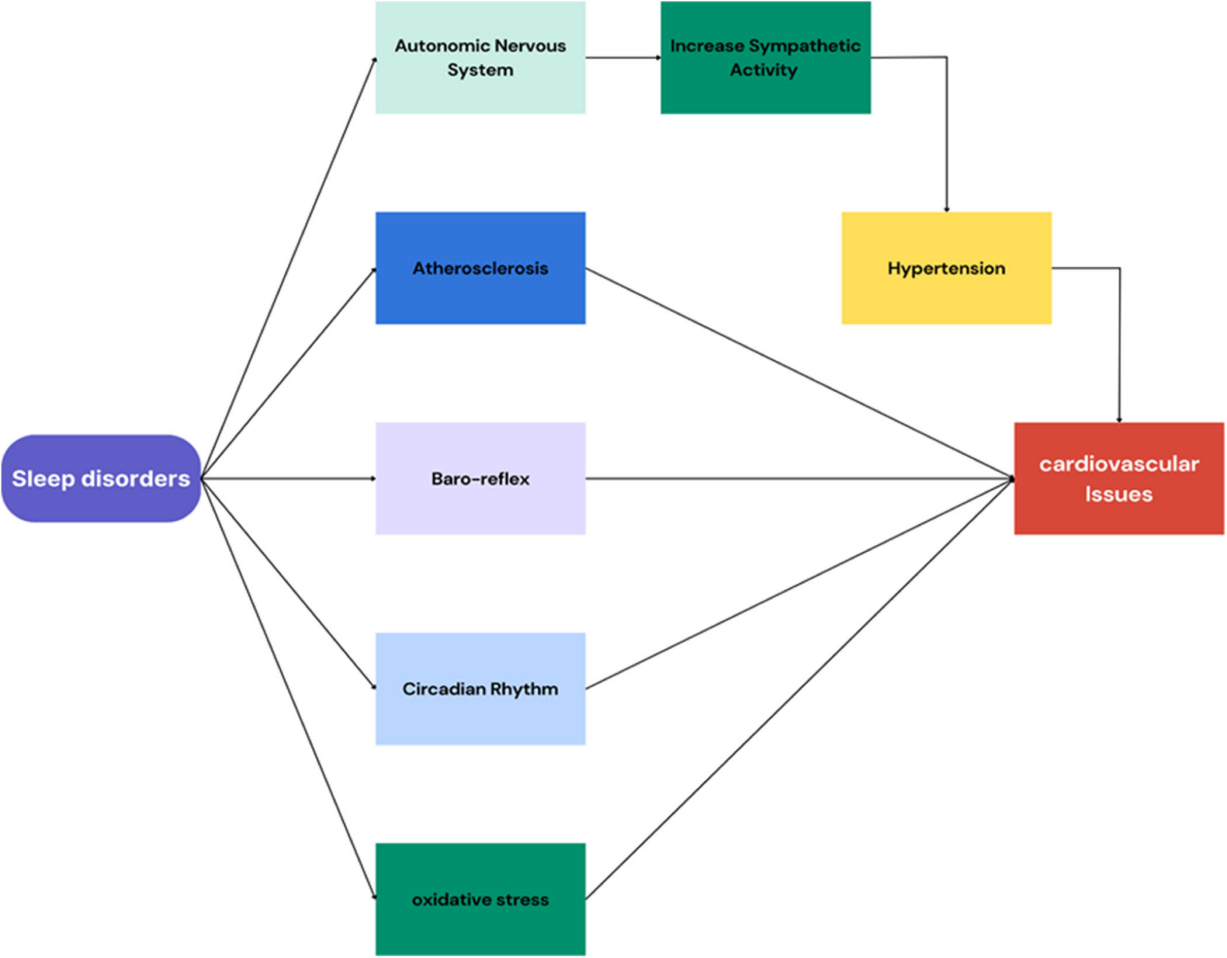
Mechanisms of Sleep Disorders' impact on cardiovascular health.

The primary cardiovascular regulating system, known as the baroreflex, is influenced by sleep‐wake cycles. We identified neurons in the nucleus of the solitary tract (NST) triggered by blood pressure elevation using activity‐dependent genetic labeling, and we verified their baro‐sensitivity. The promotion of non‐REM sleep by chemo‐ or optogenetic activation of these neurons also resulted in reduced blood pressure and heart rate. Additionally, GABA‐responsive cells in non‐REM sleep are also promoted by the caudal ventrolateral medulla (CVLM), a downstream target of the vasomotor baroreflex, in part by suppressing the sympatho‐excitatory and wake‐promoting adrenergic neurons in the brain, the rostral ventrolateral medulla (RVLM). Non‐REM sleep was also encouraged by cholinergic neurons in the nucleus ambiguous, a target of the NST for the heart baroreflex. As a result, crucial elements of the cardiovascular baroreflex circuit are also crucial for controlling the mental states of sleep and wakefulness.^31^ Correspondingly, a circadian disturbance affects patients in critical care settings and decreases the outcome following myocardial infarction. Furthermore, in heart disease models, disrupting rhythms exacerbates cardiac remodeling. Additionally, circadian desynchrony is a cause of heart disease.^32^ Sleep disturbance is linked to proinflammatory reactions, metabolic impacts, increased sympathetic nervous system activity, alterations in circadian rhythms, and the hypothalamic‐pituitary‐adrenal axis.^33^ The HPA axis’ activation and the stress‐diathesis model are just two of the various processes that could explain the link between insomnia and hypertension. It makes sense to assume that insomnia would be linked to hypertension given that research has shown that both sleep loss and short sleep duration have been linked to the condition.^34^ All antioxidants in our study were within the clinical reference range, but they were at their best in people who got enough sleep. Additionally, our study indicates that GGT is greater among the short and very short sleep duration categories, but within the clinical reference range among adequate sleepers. On the other hand, CRP is higher among the short and very short sleep duration categories but is lowest among adequate sleepers. When seen as a whole, our results imply that getting enough sleep has a positive impact on your inflammation, oxidative stress, and antioxidant profiles.^35^ Independent of the presence of traditional CVD risk factors, we found that participants with less than 6 h of sleep per night (VSSD group) have a higher burden of non‐coronary atherosclerosis and those with the most fragmented sleep (fifth FI quintile) have more affected territories as measured by 3D VUS.^36^ (Figure 2).

## 4 CLINICAL STUDIES AND FINDINGS

Sleep is essential for general health and the efficient functioning of the human body's organs and systems.^37^ However, sleep disturbances can have a negative influence on cardiovascular health. Obstructive sleep apnea (OSA) is one of the most frequent chronic conditions, along with insomnia, sleep‐disordered breathing, and restless legs syndrome.^38^, ^39^ Significant studies have linked obstructive sleep apnea to increased cardiovascular morbidity and mortality.^40^ The Sleep Heart Health Study (SHHS), a major prospective study of cardiovascular disease risk factors, included 4994 participants with an average age of 64.0 years. Participants were tracked for a median of 11.4 years using questionnaires and at‐home polysomnography (PSG). Electrocardiogram (ECG) and electroencephalogram (EEG) measurements were made using the PSG monitor. Trouble falling asleep, difficulty returning to sleep, early morning awakenings, and sleeping medication use were evaluated based on the participants’ answers in the questionnaires. This study revealed that 14.1% of 4994 participants reported insomnia or poor sleep, with 50.3% sleeping less than 6 h per night. Insomnia and short sleep (<6 h/night) combined, but not insomnia or short sleep duration alone, was significantly associated with a 29% increased risk of developing cardiovascular disease (CVD).^41^, ^42^ Despite this variation, cardiovascular outcomes and the severity of sleep disorders are linked. Another significant study is the Copenhagen City Heart Research (Østerbroundersøgelsen), a Danish prospective cohort study that surveyed a random sample of 20,000 participants aged 20–93 years. The sample was age‐stratified into 5‐year age groups, with a focus on those aged 35–70. Upon arriving at the assessment, the participants were required to fill out a questionnaire on symptoms and illnesses, medication usage, familial disposition, socioeconomic status, smoking and drinking habits, physical activity at work and in leisure time, and contact with the healthcare system. Participants consented to the study scientists retaining frozen serum and plasma and inquiring about examination findings and treatments in the healthcare system. The examination was separated into three phases, each of which was divided into stations where various vital functions such as blood pressure, fibrinogen, and pulse wave velocity tests were assessed. Following up on all‐cause mortality, the study discovered that individuals who slept for less than 6 h each night had an increased chance of developing coronary artery disease (i.e., atherosclerosis).^43^ Clinical sleep medicine relies on “cutoffs” or threshold values of a particular parameter (i.e., the apnea–hypopnoea index [AHI]).^44^, ^45^ The use of the apnea‐hypopnoea index as the primary diagnostic or prognostic tool for the diagnosis and severity grading of obstructive sleep apnea is vital.^45^ Currently, OSA's severity is mostly defined by the AHI, which quantifies the number of breathing cessations (apneas) or decreases (hypopnoeas) per hour of sleep. Mild OSA is characterized as 5–15 occurrences per hour, moderate OSA as 15 to 30 events per hour, and severe OSA as 30 or more events per hour. These categories, however, are not perfect. They are based on expert agreement rather than evidence on clinical outcomes, and they only represent one component of patient sleep apnea heterogeneity.^42^ While current research on the sleep‐cardiovascular relationship is uplifting, it is critical to be cognizant of potential confounding variables and study limitations. Bias may emerge throughout the research design, patient enrollment, data collection, analysis, and reporting stages.^46^ Confounding factors such as age, gender, ethnicity, lifestyle, medications, socioeconomic status, and comorbidities may cloud the association between sleep and cardiovascular health. The study design, data collection, and analysis can all introduce bias, thus reducing the reliability and generalizability of the findings.^47^ (Table 2).

**Table 2:** Table 1 shows key studies and findings related to sleep disorder and cardiovascular health as well as confounding factors and limitations in the current research.

Subpart	Main idea	Description	Reference
A ‐ Key studies investigating the impact of sleep disorders on cardiovascular health	‐ *Study 1:*	*Study sample:*	
	Sleep Heart Health Study (SHHS)	Study 1: 4994 participants	[41, 42]
	‐ *Study 2:*	Study 2: 20,000 participants	[43]
	Copenhagen City Heart Research (Østerbroundersøgelsen)	*Common result:* When compared to control subjects, people who slept less (<6 h/night) had a greater risk of cardiovascular disease.	[41, 42, 43]
B ‐ Findings related to cardiovascular outcomes and sleep disorder severity	Apnea–hypopnea index (AHI) remains essential in measuring sleep disorder (i.e., OSA) severity	*Mild:* 5–15 occurrences per hour	
		*Moderate:* 15–30 events per hour	
		*Severe:* 30 or more events per hour	[42]
C ‐ Potential confounding factors and limitations in the current research	Unconscious bias can interfere with the research's results.	*Confounding factors:* age, gender, lifestyle, ethnicity, drugs, socioeconomic position, and comorbidities.	[46, 47]
		*Limitations:* study design, data collecting, and analysis.	

## 5 CUTTING‐EDGE DIAGNOSTIC TOOLS FOR SLEEP DISORDERS AND CARDIOVASCULAR ASSESSMENT

In the present day, there is an upsurge in the development of sleep‐related technology for both clinical and research purposes. These advanced technologies are developed aiming at smaller‐sized devices, better scalability, affordability, mobile, and non‐obtrusive and objectively obtaining representative data even outside the laboratory in a free‐living environment.^48^

For Instance, to solve some of the drawbacks of PSG, an Ambulatory PSG is developed that uses fewer sensors and enables monitoring outside of the lab, at home. Additionally, advances in telemedicine, signal processing, and AI have made automatically scored and home VSG possible. Bed sensors have also been developed to track valuable sleep metrics such as body movement, breathing, and cardiac activities. Wireless EEGs, miniaturized EEG, and in‐ear EEG have shown promising results compared to the conventional EEG, which is part of PSG. Ultrasound sensors, WiFi, and radio‐signal approaches have also been shown to be applied for collecting physiologic signals.^48^

Moreover, Consumer sleep technologies (such as smartphone apps) have the potential to produce datasets that are exponentially larger than traditional methods, thus helping us understand the importance of sleep in health and disease.^49^, ^50^ There are smartwatches, fitness trackers, and mobile phones with different types of built‐in sensors, including microphones, gyroscopes, and accelerometers, to monitor sleep patterns.^48^

Additionally, telemedicine technologies are developed to wirelessly transmit sleep and related biosignals from a patient to a remote monitoring center. This enables recording with fewer cables, improved patient comfort, and self‐applicability, both in real‐world settings and laboratory.^49^

Furthermore, studies are being conducted to evaluate the applicability of biomarkers to the diagnosis of sleep disorders. For example, the biomarkers identified for diagnosis of insomnia were polysomnography‐derived cyclic alternating pattern, actigraphy, and BDNF levels, heart rate around sleep onset, deficient melatonin rhythm, and neuroimaging patterns (mainly for the activity of frontal and prefrontal cortex, hippocampus, and basal ganglia).^51^

In addition, there are newly emerging early markers of atherosclerosis due to sleep disorders that can alert healthcare providers to the need for intervention. These subclinical markers include prevalent Coronary artery calcium (CAC), prevalent Carotid Plaque presence, abnormal cIMT (carotid intima, media thickness), and abnormal ABI. Increased inflammatory markers (CRP, IL‐6) in individuals were also seen with irregular sleeping patterns.^52^, ^53^ Sleep disturbances, particularly the difficulty of maintaining sleep and early morning awakening, are related to higher levels of CRP.^46^

## 6 TREATMENT APPROACHES

The multimodal method of cognitive behavioral treatment for insomnia (CBT‐I) incorporates both behavioral (such as sleep restriction, stimulus control, and relaxation therapy) and cognitive (such as cognitive restructuring of dysfunctional beliefs and perceptions about sleep) strategies. CBT‐I has long‐lasting effects on insomnia and does not have the adverse side effects of hypnotic drugs. The results of studies on insomnia that have assessed biomarkers support the potential advantages of CBT‐I in preventing the onset of CVD or a worsening of the condition through its metabolic, inflammatory, autonomic, and circadian effects. As a result, it suggests a promising method to improve functional capability and quality of life while reducing cardiovascular morbidity and symptom burden.^54^

Continuous positive airway pressure (CPAP) use has been shown to improve blood pressure, cognition assessment, sleep‐related symptoms, anxiety, and depression compared to no treatment. Given that obesity is a frequent feature in many research, it is challenging to thoroughly evaluate the impact of CPAP on cardiovascular alterations related to OSA.^55^

Short‐term CPAP treatment has demonstrated a small decrease in blood pressure, as well as improvements in some other CV risk indicators, although not all intervention studies have been favorable. Larger, longer‐followed randomized controlled trials have validated a modest BP decreases with CPAP.^56^

According to a systematic review which included 15 researches, the possibility of medication help for hospitalized patients with poor sleep is not supported by enough evidence.^57^ However, it was shown in a rat model study that 10 mg/kg of α‐Asarone, improved the quality of sleep, as indicated by an increased NREM bout duration, reduced arousal index, and decreased bout frequencies of NREM sleep and wakefulness.^58^

By speeding the beginning of sleep, increasing its efficiency, and lengthening the total time, dual and selective orexin‐2 receptor antagonists have demonstrated effectiveness in causing sleep in patients with insomnia disorders.^59^ On the other hand, a systematic review advises against using Z‐drugs, for treating insomnia, despite its benefit in lengthening sleep, primarily due to the significant risk of fractures.^60^

Melatonin used exogenously has positive impacts on cardiovascular health and improves the quality of sleep in patients with insomnia.^61^ Another study demonstrated that 8 weeks, oral, 3 g *Melissa officinalis* can decrease sleep disorders in people with chronic stable angina.^62^

Furthermore, The combination of ischemic stroke and obstructive sleep apnea may constitute a unique phenotype that responds effectively to trazodone and empagliflozin by lowering the risk of new‐onset OSA, with positive benefits on CV outcomes.^63^, ^64^

**Figure 3:**
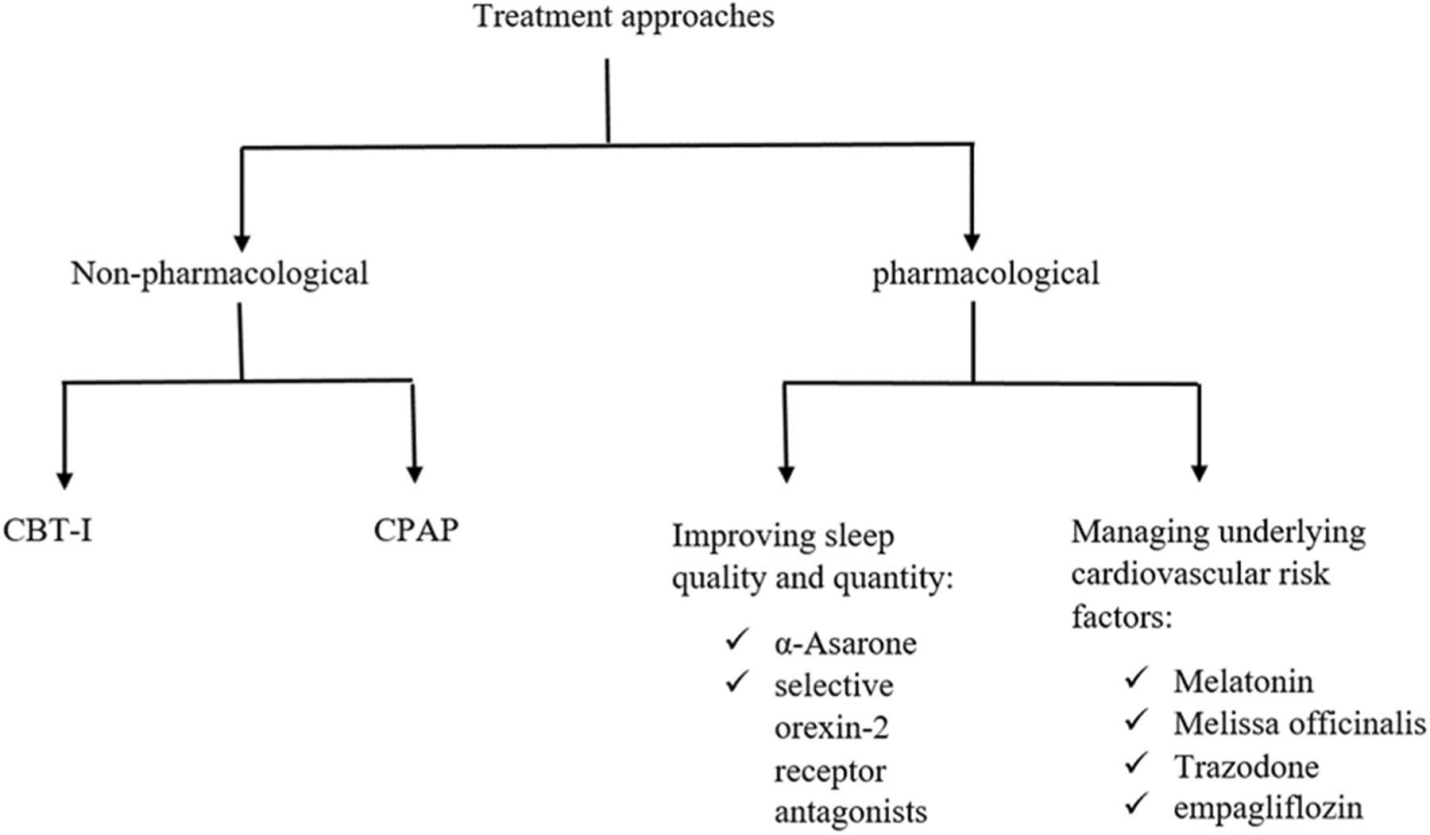
Figure represents a summary of treatment approaches for sleep disorders and its related CVD.

On the other hand, a postmenopausal woman being treated for sleep disorders, Z‐drugs were linked to increased risk of CVD.^65^ Finally, unless paired with significant weight gain in the long term, no CVD‐targeted medications significantly treat sleep apnea.^66^ (Figure 3).

## 7 FUTURE DIRECTIONS AND IMPLICATIONS

Investigation into the relationship between sleep disorders and cardiovascular health is a dynamic field, and several burgeoning research areas are gaining attention, such as Sleep heart failure, sleep hypertension and Sleep, Circadian Rhythms, and Metabolism. Exploring these emerging areas promises to deepen our understanding of the complex relationship between sleep and heart health, potentially leading to novel interventions and improved patient care. Personalized medicine is the process of assessing various aspects of an individual patient, including their genetics, biochemistry, and behavior, to gain insights into how they might respond to a specific treatment. This approach is crucial due to the presence and ongoing discovery of significant differences in how individuals respond to treatments. Modern biomedical tools like DNA sequencing, proteomics, and wireless monitoring devices have made it possible to identify these variations, underscoring the necessity to personalize medical interventions to some degree.^67^ Collaboration between electrophysiologists/cardiologists and sleep specialists is essential for incorporating sleep‐disordered breathing testing and treatment into atrial fibrillation (AF) clinics. This collaboration works best within a unified care approach to enhance comprehensive healthcare and avoid fragmentation. Effective partnership among sleep physicians, companies, nurses, patients, and cardiologists/electrophysiologists is necessary to create efficient and economical patient pathways that prioritize convenience.^68^

## 8 CONCLUSION

This study emphasizes sleep's influence on cardiovascular health, highlighting the dangers of insufficient sleep and sleep disorders such as insomnia, sleep apnea, and so on. The relationship between sleep patterns and cardiovascular well‐being requires close attention, and emerging diagnostic tools like Ultrasound sensors, biomarkers, smart watches, accelerometers, and so on, promise concrete improvement. Treatment benefits from cognitive therapy to CPAP have been identified. Personalized interventions and collaborative cross‐disciplinary efforts remain essential. Distinguishing the clinical role of sleep among the public can pave the way for improved cardiovascular outcomes. Also, there is a need to explore molecular pathways linking sleep and cardiovascular health.

Collaboration and a multidisciplinary strategy can effectively bridge these gaps. Technological support, advanced research mechanisms, and integrating policies can further improve sleep‐cardiovascular outcomes.

## AUTHOR CONTRIBUTIONS

**Christin Berjaoui**: Supervision; Visualization; Writing—original draft; Writing—review & editing. **Bethlehem Tesfasilassie Kibrom**: Writing—original draft; Writing—review & editing. **Mohammad Ghayyad**: Writing—original draft; Writing—review & editing. **Safaa Joumaa**: Writing—original draft; Writing—review & editing. **Nihal Talal Al Labban**: Writing—original draft; Writing—review & editing. **Abubakar Nazir**: Conceptualization; Supervision; Writing—original draft; Writing—review & editing. **Charbel Kachouh**: Writing—original draft; Writing—review & editing. **Abdulrahmon Akanmu Moradeyo**: Writing—original draft; Writing—review & editing. **Magda Wojtara**: Writing—original draft; Writing—review & editing. **Olivier Uwishema**: Writing—original draft; Writing—review & editing.

## CONFLICT OF INTEREST STATEMENT

The authors declare no conflict of interest.

## TRANSPARENCY STATEMENT

The lead author Abubakar Nazir affirms that this manuscript is an honest, accurate, and transparent account of the study being reported; that no important aspects of the study have been omitted; and that any discrepancies from the study as planned (and, if relevant, registered) have been explained.

## Data Availability

Not Applicable.
